# Mechanisms of Functional Pleiotropy of p73 in Cancer and Beyond

**DOI:** 10.3389/fcell.2021.737735

**Published:** 2021-09-28

**Authors:** Stella Logotheti, Christin Richter, Nico Murr, Alf Spitschak, Stephan Marquardt, Brigitte M. Pützer

**Affiliations:** ^1^Institute of Experimental Gene Therapy and Cancer Research, Rostock University Medical Center, Rostock, Germany; ^2^Department Life, Light & Matter, University of Rostock, Rostock, Germany

**Keywords:** p73, protein–protein interactions(PPI), C-terminus, gene promoter architecture, development and homeostasis, differentiation, neoneurogenesis, cancer progression

## Abstract

The transcription factor p73 is a structural and functional homolog of TP53, the most famous and frequently mutated tumor-suppressor gene. The TP73 gene can synthesize an overwhelming number of isoforms via splicing events in 5′ and 3′ ends and alternative promoter usage. Although it originally came into the spotlight due to the potential of several of these isoforms to mimic p53 functions, it is now clear that TP73 has its own unique identity as a master regulator of multifaceted processes in embryonic development, tissue homeostasis, and cancer. This remarkable functional pleiotropy is supported by a high degree of mechanistic heterogeneity, which extends far-beyond the typical mode of action by transactivation and largely relies on the ability of p73 isoforms to form protein–protein interactions (PPIs) with a variety of nuclear and cytoplasmic proteins. Importantly, each p73 isoform carries a unique combination of functional domains and residues that facilitates the establishment of PPIs in a highly selective manner. Herein, we summarize the expanding functional repertoire of TP73 in physiological and oncogenic processes. We emphasize how TP73’s ability to control neurodevelopment and neurodifferentiation is co-opted in cancer cells toward neoneurogenesis, an emerging cancer hallmark, whereby tumors promote their own innervation. By further exploring the canonical and non-canonical mechanistic patterns of p73, we apprehend its functional diversity as the result of a sophisticated and coordinated interplay of: (a) the type of p73 isoforms (b) the presence of p73 interaction partners in the cell milieu, and (c) the architecture of target gene promoters. We suppose that dysregulation of one or more of these parameters in tumors may lead to cancer initiation and progression by reactivating p73 isoforms and/or p73-regulated differentiation programs thereof in a spatiotemporally inappropriate manner. A thorough understanding of the mechanisms supporting p73 functional diversity is of paramount importance for the efficient and precise p73 targeting not only in cancer, but also in other pathological conditions where TP73 dysregulation is causally involved.

## Introduction

TP53 is a well-known tumor suppressor and a famous “Holy Grail” of anticancer targeting. In 1997, two genes were added in the so-far considered single-membered p53 family: p63 and p73, which present similarities to p53 regarding their basic functional domains and their ability to activate typical p53 targets and participate in common p53 pathways. Nevertheless, both also differ from p53, since they do not exhibit the characteristics of a classical Knudson-type tumor suppressor gene. p63 and p73 entail three basic functional domains which are homologous to p53, that is the transactivation domain (TA), the core DNA-binding domain (DBD) and the oligomerization domain (OD), with DBD being the most conserved domain. They have an extra SAM (sterile alpha motif) domain in their C-terminus, which confers protein stability (Logotheti et al., 2013). Although *p53* appeared later in evolution, to specifically guard the fidelity of somatic cell divisions and protect from DNA damage-induced cancerous alterations, the ability to regulate DNA damage and apoptosis is primitive within the p53 family. In bony fishes, the *p63*/*p73* and the *p53* genes are separated into distinct entities and undergo positive selection to control different processes: p63 got specialized in the production of epithelial cells and p73 in neuronal development, whereas p53 is better adapted as a tumor suppressor (Belyi and Levine, 2009; Belyi et al., 2010). These functional adaptations were associated with alterations in the gene organization of a single ancestral p53/p63/p73 gene, mainly gain of an alternative promoter that leads to NH2-terminal truncated isoforms, loss of the SAM domain, and ability for formation of additional C-terminal splice variants in *Chordata* and evolutionarily higher organisms (Yang et al., 2002). These changes overall enabled a higher level of functional divergence and specificity for each p53 family member. All three family members synthesize many isoforms, with p73 producing the highest number.

**FIGURE 1:**
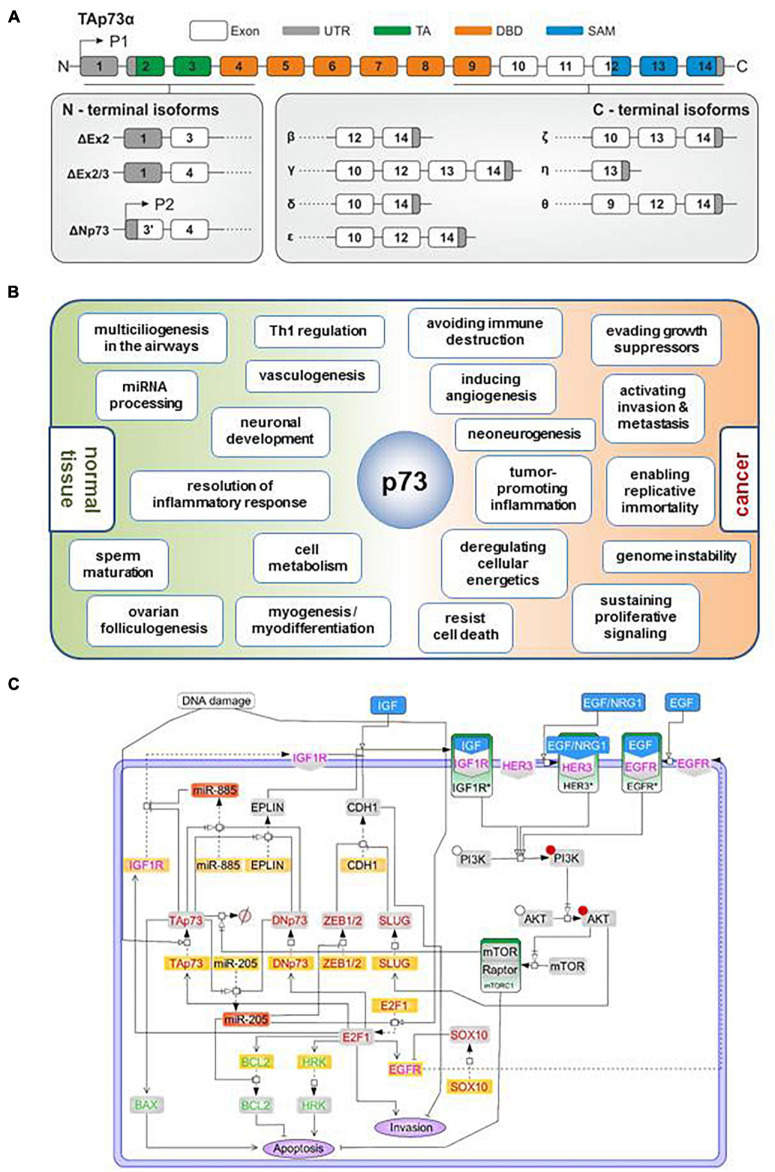
TP73 synthesizes a large number of isoforms contributing to its multifunctionality both in physiological and cancer-related processes. **(A)** Diagram depicting synthesis of p73 isoforms by (i) alternative splicing in the 3′ end, putting forth several C-terminal splice variants; or (ii) the use of an extrinsic (P1) and alternative intrinsic promoter (P2) in the 5′ end, and (iii) alternative splicing in the 5′ end. All isoforms contain a core DNA-binding domain. Different combinations of the N-terminal head with the C-terminal tail give rise to functionally distinct isoforms. TA, transactivation domain; DBD, DNA-binding domain; SAM, sterile alpha motif. **(B)** Overview of the documented and predicted roles of TP73 gene products in normal and cancer tissues. There are analogies in several processes regulated by TP73 in the physiological and cancer context (terms straddling around the p73 node in the scheme) which might represent the aberrant function of p73 regulatory networks of embryonic development, differentiation, and tissue homeostasis within the tumor context. **(C)** Regulatory map of the TAp73/DNp73-controlled pathways depicting their interception with major cascades of extracellular signaling. The model was constructed using SBGN-compliant software Cell Designer. Yellow boxes: RNA; gray boxes: protein; red boxes: mature microRNAs; green boxes: activated signaling complexes; blue boxes: receptor ligands; red font: transcription factors, green font: apoptosis related proteins; magenta font: growth factors. Phosphorylated proteins are marked with a red circle.

The synthesis of this overwhelmingly large number of p73 isoforms is achieved by (a) use of an extrinsic (P1) and alternative intrinsic promoter (P2) in the 5′ end, generating TA and Δ*N* classes of isoforms, (b) alternative splicing in the 5′ end, resulting in amino-truncated ΔTA isoforms (ΔEx2p73, ΔEx2/3p73, and ΔN′p73) that partially or entirely lack the transactivation domain and, together with Δ*N*, constitute the so called DN isoforms, (c) alternative splicing in the 3′ end, putting forth several C-terminal splice variants (α, β, γ, δ, ε, ζ, η, η^∗^, η1, and θ) (Logotheti et al., 2013) (Figure 1A). We also detected somatic genomic rearrangements of TP73 that generate an oncogenic TP73ex2/3 (George et al., 2015). In the context of cancer, TAp73 isoforms are considered anti-oncogenic (Stiewe and Pützer, 2000; Malik et al., 2021), while DNp73 forms antagonize TAp73 effects and are oncogenic. The opposing roles of TA versus ΔN classes of isoforms in the context of cancer have been underscored via knockout mice that are selectively deficient for either TAp73 or ΔNp73. TA p73-knockout mice are tumor-prone and more sensitive to carcinogens, and present genomic instability and enhanced aneuploidy, thus highlighting a significant role of TAp73 in the maintenance of genomic integrity (Tomasini et al., 2008). In contrast, mice lacking ΔN p73 show increased apoptosis in response to DNA damage, revealing an oncogenic effect of this isoform in the inhibition of the DNA-damage response (DDR) pathway (Wilhelm et al., 2010). Using kinetic modeling to detect genetic signatures characteristic for cancer drug resistance, we have shown for example that downregulation of miR-205 can be mediated by an imbalance in the TAp73/ΔTA ratio, which leads to increased expression of anti-apoptotic BCL-2 and ABC transporters (Alla et al., 2012; Vera et al., 2013). The ΔTA’s that are tumor-specific, are vigorously expressed in advanced stages across a variety of highly aggressive human cancers (Knoll et al., 2011; Steder et al., 2013; George et al., 2015; Meier et al., 2016) and, analogous to Δ*N*’s, promote tumor initiation (Tannapfel et al., 2008) and cancer progression (Engelmann et al., 2015). For instance, DNp73 (p73ΔEx2/3) is a key metastatic driver, which promotes tumor progression via inducing EMT, actin cytoskeletal reorganization, invasion, and stemness in melanoma (Steder et al., 2013; Engelmann and Pützer, 2014; Meier et al., 2016; Fürst et al., 2019). Consistently, p73 isoforms play a key role in the regulation of cancer stemness, epithelial-mesenchymal transition (EMT) and response to therapy across several cancer types (Prabhu et al., 2016; Thakur et al., 2016; Sharif et al., 2019b; Uboveja et al., 2020). For a detailed overview, we refer to Engelmann et al. (2015), Pützer et al. (2017).

The TP73 gene has attracted incredible attention for therapeutic cancer management mainly because it can mimic and/or surrogate for p53 oncosuppressive functions, whereas unlike p53, it is rarely mutated in cancer, a fact that renders this targeting unbiased from intra- and inter-tumoral mutational heterogeneity (Logotheti et al., 2019). Hence, understanding the mechanisms through which p73 isoforms exert their functions are of paramount importance in order to efficiently manipulate these factors in precision medicine. All TA and DNp73 isoforms have intact core DNA-binding and tetramerization domains, via which they can oligomerize and bind to corresponding p53- or p73-responsive elements (RE). The transactivation domain-containing TA’s directly activate the transcription of p53/TAp73 target genes. Vice versa, DNp73 lacking a typical N-terminal transactivation domain can act as transdominant inhibitors of TAp73 and p53 (Stiewe et al., 2002a,b) and block their gene regulatory activity either by competing for p53/p73 binding sites or by forming transcriptionally silent TAp73/DNp73 or p53/DNp73 hetero-oligomers (Marabese et al., 2007). The ultimate effect of p73 isoforms on target genes is overall attributed to the TA/DN ratio as opposed to the overexpression of a specific p73 isoform or a specific class of p73 isoforms *per se*. Furthermore, the p53/p63 cell content often influences p73 functions, because p73 can form stable hetero-oligomers with p63 or mutant p53 (Li and Prives, 2007; Nemajerova et al., 2018).

p73 has been initially viewed as a p53 ‘copycatter,’ because of its ability to activate common p53 targets and regulate tumor-suppressive processes, such as cell cycle arrest, senescence, apoptosis and genomic stability. However, it has currently been realized that p73 not only affects a large number of cancer-related pathways, but also regulates disparate processes in embryonic development and tissue homeostasis. More intriguingly, p73 appears to have the potential to activate ‘off-context’ its non-oncogenic differentiation programs within the cancer cell context to modulate tumor metastasis. This process, whereby a biological function within a specific context may be alternatively used in another context to support a novel function, is termed co-option and consists a recurrent and prevailing pattern during tumor progression (Billaud and Santoro, 2011). The high level of functional pleiotropy in physiological and cancer-related processes cannot be sufficiently supported solely by the typical mode of direct gene transactivation/transrepression that has been described for p73, but rather suggests more sophisticated mechanisms of action of p73 in multiple levels of gene regulation. This review summarizes the roles of p73 isoforms in physiological and oncogenic processes and describes how its ability to control development and/or differentiation can be hijacked during cancer progression and within the tumor microenvironment (TME). It further sheds light on the mechanistical patterns governing its disparate functions by using the melanoma setting as a paradigm and suggests that a sophisticated and highly coordinated interplay between the p73 C-terminus, the protein interactome, target gene promoter architecture (Rudge et al., 2016) and the subcellular localization of TP73-derived isoforms can support this multifunctionality.

## The Expanding Functional Repertoire of Tp73 in Cancer and Beyond

Besides their well-demonstrated roles in DDR and apoptosis (Wilhelm et al., 2010), p73 isoforms also have unique targets (Logotheti et al., 2019; Wang et al., 2020) and exert non-oncogenic functions (Inoue et al., 2014) that are not shared with p53. Understanding their functional pleiotropy has been enabled through the use of DNp73 splice isoform-specific antisense oligonucleotide gapmers (Emmrich et al., 2009) and by knockout mice models with (i) deletion of the entire TP73 gene, (ii) deletion of exons encoding the TAp73 isoforms, (iii) deletion of exons encoding the ΔNp73 isoform, and (iv) deletions of exons encoding the C-terminus of the alpha isoform. These tools in combination with expression and molecular studies, have allowed to uncover the roles of TP73 in cancer and beyond, such as neurodevelopment, ciliogenesis, and metabolism (Melino, 2020). In this chapter, we summarize the non-oncogenic as well as the cancer-related functions of p73 isoforms. We also report that p73 has newly identified roles in regulating the neurogenic potential of tumors by co-opting, in a cancer cell context, its neurodevelopmental/neurodifferentiation programs (Logotheti et al., 2020b).

### Roles in Development, Differentiation and Tissue Homeostasis

The generation of the first p73 KO mice unambiguously showed that deletion of all p73 isoforms creates a wide range of neurological, pheromonal and inflammatory defects (Yang et al., 2000). A recurring and predominant phenotypic outcome upon ablation of either global (pan-TP73KO) or isoform class-specific (TAp73 or ΔNp73 KO) knockdown of TP73 products is manifestation of several neurodevelopmental abnormalities. In particular, pan-TP73KO mice display severe hydrocephalus and hippocampal dysgenesis characterized by partial or total loss of the lower blade of the dentate gyrus (DG) and by an impaired organization of CA1 and CA3 regions. TAp73KO mice show a less severe phenotype, but still exert abnormal hippocampal development, whereas ΔNp73 mice demonstrate only marginal reduction of cortical thickness but no hydrocephalus (Nemajerova et al., 2018). In general, TAp73 is required for neuronal differentiation and maintenance of neural stem cells (NSCs), while ΔNp73 is needed for neuronal survival during development and in adult neuronal tissues (Killick et al., 2011). In addition, p73 is essential for maintaining the neurogenic pool in the subventricular (SVZ) and subgranular zone (SGZ) through promoting self-renewal and proliferation, and inhibiting premature senescence of NSCs and/or neural precursor cells (NPCs). Mechanistically, TAp73 either directly or indirectly regulates the expression of genes involved in NSCs maintenance (Sox2, Sox3, TRIM32, and Hey-2), in axonal growth and dendritic arborisation (neurotrophin receptor p75), and in neuronal terminal differentiation (synaptotagmin-1 and syntaxin-1A). The recently described Trp73^d13/d13^ mice, lacking exon 13 in the p73 gene, have revealed a significant contribution of the C-terminus to the brain development. Deletion of exon 13 produces a switch of the longest and most expressed isoform α into β, which in contrast to α lacks the SAM domain. Replacement of α with β substantially affects brain development, producing hippocampal dysgenesis, in particular, progressive depauperation of Cajal-Retzius (CR) cells in the developing brain. Hippocampal dysgenesis appears to be a consequence of deprivation of CR cells that are physiologically deputed to direct brain architecture during embryonic development. These effects appear to be highly isoform-dependent (Killick et al., 2011). Hence, not only the N-terminus of p73 isoforms, but also in their carboxy-terminal sequences have divergent effects on neuronal differentiation, maintenance of NSCs, neuronal survival during development and in adult neuronal tissues (Killick et al., 2011). Overall, p73 is nodal in the regulation of CNS development and function, by modulating NSC self-renewal and differentiation and by promoting terminal neuronal differentiation (Niklison-Chirou et al., 2020). The fact that these phenotypes are non-overlapping with the neurodevelopmental defects caused by the deletion of all p73 isoforms (p73 KO mice), the TAp73 isoforms (TAp73 KO) or the ΔNp73 (ΔNp73 KO mice), indicates indispensable, yet distinct, roles of the N-terminal, core and C-terminal domains of p73 in the regulation of neuronal processes.

In addition to their prevalent role in the central and peripheral nervous system, p73 isoforms have tissue-specific roles in the male and female reproductive organs, the development of respiratory epithelium and the vascular network. A recently discovered function of p73 manifested across several tissues is the differentiation and fate specification of multiciliated cells (MCC), which are vital for respiration, neurogenesis and fertility. Moreover, TAp73 shows a significant ability to regulate cellular metabolism. The abovementioned physiological functions of p73 in these organ systems have been extensively reviewed elsewhere (Nemajerova et al., 2018; Nemajerova and Moll, 2019; Maeso-Alonso et al., 2021). Besides these roles, p73 isoforms are essential for the proper function of the immune system. On one hand, TAp73 is required for macrophage-mediated innate immunity and the resolution of inflammatory response. TAp73 KO alters macrophage polarization such that maintenance of the M1 effector phenotype is prolonged at the expense of the M2 phenotype, thus impairing resolution of the inflammation (Tomasini et al., 2013). On the other hand, regarding adaptive immunity, Ren et al. (2020) recently identified p73 as a negative regulator of the Th1 immune response via transrepression of IFN gamma transcription and downregulation of IFN gamma production.

Last but not least, several lines of evidence imply a key role of TP73 protein products in the differentiation and homeostasis in several types of muscle tissues. In skeletal muscles, TAp73α but not TAp73β isoform suppresses myogenic differentiation (Li et al., 2005), while ΔNp73α protect differentiated myotubes from DNA damage-induced apoptosis and inhibits the spontaneous apoptosis of satellite skeletal muscle cells that fail to complete their differentiation. Upregulation of the p73 P2 promoter during myogenic differentiation is mediated by a coordinated recruitment and activity of p53/p73 and the master-regulator of muscle cell development, MyoD (Belloni et al., 2006). In smooth muscles, p73 induces apoptosis of vascular smooth muscle cells and is present at high levels in human atherosclerotic plaque (Weiss and Howard, 2001; Davis et al., 2003). In the cardiac tissue, low expression of TP73 products has been observed in cardiomyocytes and other cell types of the heart muscle (data mined from Human Protein Atlas), while others have shown that the directed expression of DNp73 stimulates proliferation of cardiomyocytes via antagonizing p53 (Ebelt et al., 2008). Basal p53 levels are essential for embryonic cardiac development and for maintaining normal heart architecture and physiological function (Men et al., 2021), but it has not been investigated whether p73 isoform(s) participate in these processes. Based on these hints, it would be interesting to explore a so far unnoticed physiological role for p73 isoforms in cardiac development and physiology, a possibility that would further pave new avenues in treatment of cardiovascular diseases and/or cardiac tissue regeneration. The functional diversity of p73 isoforms in embryonic development, differentiation, and tissue homeostasis is depicted in Figure 1B. It has been proposed that a common unifying theme among several seemingly divergent p73-regulated physiological functions is that p73 acts as a master-regulator of tissue architecture, and that such a role might have been inherited from a single p53/p63/p73-hybrid gene ancestor at the dawn of epithelial tissue evolution, which is traced back to Placozoans and Cnidaria (Maeso-Alonso et al., 2021).

### p73 Involvement in Cancer Hallmarks and Oncogenic Signaling Cascades

Oncogenic transformation occurs through progressive acquisition of key adaptations, the so-called cancer hallmarks, which include sustaining proliferative signaling, resisting cell death, evading growth suppressors, activating invasion, enabling replicative immortality, inducing angiogenesis, reprogramming energy metabolism and evading immune destruction. Transformation is further facilitated by tumor-promoting inflammation and genome instability. Strikingly, TAp73 isoforms inhibit all these hallmarks [reviewed in detail in Logotheti et al. (2013)] and can also enhance responsiveness to standard radio- and chemotherapies (Logotheti et al., 2013) (Figure 1B). Their DNp73 counterparts can typically antagonize these functions, thereby drastically influencing cancer promotion, progression and metastasis [reviewed in Logotheti et al. (2013), Engelmann and Pützer (2014), and Engelmann et al. (2015)]. Recently, it was shown that TAp73 regulates macrophage accumulation and tumor infiltration, which is in general a strong driver of cancer progression and predictor of poor outcomes in cancer patients. This occurs via inhibition of the NF-κB pathway, since loss of TAp73 leads to NF-κB hyperactivation and secretion of Ccl2, a known NF-κB target and chemoattractant for monocytes and macrophages. Importantly, TAp73-deficient tumors display an increased accumulation of protumoral macrophages that express the mannose receptor (CD206) and scavenger receptor A (CD204) (Wolfsberger et al., 2021).

It is noteworthy that in some cancer-related processes and cell-contents, TAp73 isoforms show an effect inconsistent with their traditional tumor suppressive function. For example, TAp73 activates anabolic pathways compatible with proliferation and cancer promotion by regulating glucose metabolism to control cellular biosynthetic pathways and antioxidant capacity [reviewed in Nemajerova et al. (2018)]. TAp73 modifies the metabolism and positively regulates growth of cancer stem-like cells in a redox-sensitive manner (Sharif et al., 2019a). Nevertheless, it remains still unclear whether this metabolic effect reflects cancer-associated metabolic changes, or instead a role in promoting adaptative cellular mechanisms to stress conditions [reviewed in Nemajerova et al. (2018) and Maeso-Alonso et al. (2021) and]. Furthermore, p73 proteins regulate angiogenesis, with the ΔNp73 form that has a clear role in promoting this phenomenon, whereas TAp73 isoforms exert both, positive and negative effects, depending on parameters like the strength and spatiotemporal context of its activation (Sabapathy, 2015). It is also not clear if every TAp73 isoform can exhibit such Janus behavior in metastasis-promoting processes, or if this is an attribute of only specific TAp73 C-terminus splice variants.

Using our previous work on melanoma as a representative example of p73’s regulation of cancer outcomes via orchestrating molecular networks, we further made the striking observation that TAp73/DNp73-controlled pathways can co-ordinate extracellular changes in the TME and intracellular gene regulation by intercepting with major cellular signaling cascades which respond to growth factors in the TME. These intersections suggest that p73 isoforms internalize the information from extracellular signals that are received by cell surface receptors and convey them to the nucleus, leading to global changes in p73-transcriptome. In particular, we used the melanoma paradigm and constructed a comprehensive regulatory map by exploiting own high-throughput and experimental data from melanoma tissue culture, mouse metastasis models and patient tumor samples. These results were integrated with data from the literature, previous mathematical models describing sections of the map (Vera et al., 2013; Khan et al., 2014), and partial elements of existing IGF1R computational models (Borisov et al., 2009; Bianconi et al., 2012). The reconstructed network demonstrates that TAp73/DNp73-dependent pathways (Alla et al., 2010, 2012) intercept with cellular receptor-triggered signaling cascades that are relevant for melanoma progression, such as EGFR (Sun et al., 2014; Wang et al., 2015), IGFR (Rosenbluth et al., 2008), and HER3 (Iorio et al., 2009; Tiwary et al., 2014) and portray the potential of p73 isoforms to sense changes in the cell microenvironment and modify the gene regulation programs accordingly (Figure 1C).

### Regulation of Cancer Neurobiology – p73 and the Emerging Hallmark of Neoneurogenesis

Of particular interest is that neurodevelopmental defects and cancer-related phenotypes co-exist in TAp73 knockout and, to a much lesser extent, in ΔNp73 KO mice. The ability of p73 isoforms to modulate tumor initiation and progression may be relevant to their neurological functions. In particular, it is becoming increasingly evident that cancer and neuronal cells develop reciprocal interactions via mutual production and secretion of neuronal growth factors, neurothrophins and/or axon guidance molecules in the TME. Intriguingly, tumors can stimulate their own innervation during cancer progression, and this phenomenon is termed neoneurogenesis. Tumors produce and excrete neurogenic factors that modulate the TME and induce formation of new nerves that eventually infiltrate tumors (Logotheti et al., 2020b). Recently, Mauffrey et al. (2019) showed that prostate tumors summon neural progenitors from sites as distant as the subventricular zone of the central nervous system (CNS), which break the blood-brain barrier, infiltrate prostate tumors and initiate neurogenesis. This process is essentially distinct from perineural invasion (PNI), which refers to tumor invading into already existing nerves along the perineural space. Besides, cancer cells themselves may acquire brain-like properties as an adaptation for brain colonization (Neman et al., 2014). The nervous system-cancer crosstalk emerges as a crucial regulator of cancer initiation and progression, both systemically and within the local TME. In turn, cancers and cancer therapies can alter nervous system form and function Tumors may induce profound nervous system remodeling and dysfunction by secreting circulating factors which not only locally alter neural activity in the TME, but also have a remote and systemic effect on important brain functions, such as sleep. The interactions between neural and malignant cells are highly relevant for cancer clinical therapy, since they are suspected to be involved, at least in part, in neuronal toxicities induced by radiation and chemotherapies (Monje et al., 2020). The mechanisms that support this enigmatic crosstalk between tumors and the nervous system remain largely unexplored. We have recently provided mechanistic insights that the neurogenic potential of tumors appears to be induced, at least in part, by co-option of neuronal processes within cancer cells. Genes involved in neuronal development and function are reactivated in various tumor types and predict poor patient outcomes. The ectopic activation of neuronal programs in cancer cells and the switch to neurogenic phenotypes does not appear to be a stochastic, random event, but rather provides selective advantages to tumor cells (Logotheti et al., 2020b). Moreover, deregulation of genes that are indispensable for nervous system development and neurological function are associated with long-term survival in adult AML [Yılmaz et al., 2021), accepted for publication]. In this regard, neoneurogenesis might constitute a novel cancer hallmark, comparable to angio- and lymphangiogenesis.

It is possible that the ability of p73 isoforms to regulate several cancer hallmarks may not be independent from their neurological and/or immunomodulatory functions, but instead might imply co-options of relevant p73-governed pathways in a cancer cell context. Looking at the processes regulated by p73 isoforms in normal and cancerous tissues more closely, analogies can be found between some physiological processes and cancer hallmarks. It is well-accepted that ΔNp73 overexpression becomes a positive advantage for tumor progression due to co-option of its pro-angiogenic capacity in tumors that trigger neoangiogenesis (Nemajerova and Moll, 2019). In an analogous manner, it is reasonable to assume that recapitulation of the same p73-regulated neurodevelopmental/neurodifferentiation pathways in cancer cells could promote tumor progression, for example by inducing neoneurogenesis or by altering interactions of cancer cells with neuronal and immune cells (Figure 1B). Several p73 isoforms may positively or even negatively regulate cancer invasion and metastasis through activating their nervous system-related target genes within the cancer cell context. In support of this rationale, TAp73 isoforms control cancer cell proliferation, migration and invasion through transactivation of the brain-enriched miRNA gene MIR3158, which targets vimentin (Galtsidis et al., 2017). Similarly, ΔTAp73 (p73ΔEx2/3 α and β) expression in less-invasive melanoma cells enhances stemness and self-renewal capacity through an interplay with MIR885 (Meier et al., 2016), a miRNA with brain/cerebellum-restricted expression [data mined from miRiad database (Hinske et al., 2014)], that targets IGFR (Meier et al., 2016). Again in an IGFR-dependent manner, p73ΔEx2/3 drives EMT phenotypic conversion and initiation of metastasis in melanoma (Steder et al., 2013), along with tyrosinase degradation, depigmentation and loss of melanocyte identity (Fürst et al., 2019). In the presence of p73ΔEx2/3 and persistently high TAp73α levels, melanoma cells lose their original cell-type characteristics, and simultaneously activate stemness (Meier et al., 2016), EMT (Steder et al., 2013), and nervous system-related genes. Upregulation of stemness markers in aggressive melanoma states is accompanied by increased expression of key neurotrophic factors, including BDNF, which was recently shown to foster neoneurogenesis (Logotheti et al., 2020b).

In view of these data, our results suggest that p73 isoforms co-regulate stemness and neurodifferentiation to control tumor progression. The tumor-specific p73ΔEx2/3 isoforms, which are established metastasis inducers and CSC regulators, have the ability to activate key neurodifferentiation players. Increased cancer stemness, together with the loss of original cell identity (de-differentiation), and the acquisition of characteristics of neuronal cell types upon p73 isoform expression, are overall indicative for their ability to switch from a melanocyte to a neuronal-like cell phenotype which would be theoretically able to foster a newly-emerged dangerous liaison between melanomas and the nervous system (Su et al., 2014; Lu et al., 2017; Logotheti et al., 2020b; Pomeranz Krummel et al., 2021).

## Dissecting the Functional Pleiotropy of Tp73

In light of the overwhelming functional diversity of the products of TP73, it is reasonable to assume that the typical mode of direct transactivation/transrepression that has been described for TAp73 and DNp73’s, on its own, is not sufficient to support such high level of functional pleiotropy across several organ systems. Besides the TA/DN ratio, isoforms synthesized by other members of the p53 family, like p53 and p63, also influence p73 activity (Li and Prives, 2007; Nemajerova et al., 2018). However, even distinct p53/p63 backgrounds, wildtype or mutant, cannot explain the plethora of p73 effects in different cellular contexts. The divergent and tissue-specific roles of p73 imply a high degree of complexity and sophistication in relation to its modes of function. Several lines of evidence instead argue for a possible existence of p73 traits complementary to its canonical function as a transcription factor. First of all, global genomic binding studies (Rosenbluth et al., 2008; Koeppel et al., 2011) indicate that only a disproportionally small fraction of p73-responsive genes directly binds p73 to induce p73-mediated functional changes. Second, the ΔNp73 isoforms bear a unique 13-amino acid motif in their N-terminus that possesses transactivation potential (Liu et al., 2004), questioning the dogma that ΔNp73’s are transcriptionally inactive at all times. Third, there is a certain degree of controversy, whereby in some cellular contexts, TAp73 isoforms might regulate anti-apoptotic and pro-survival genes (Wang et al., 2020) and ΔNp73 isoforms can activate apoptotic targets (Liu et al., 2004; Toh et al., 2008), a fact that pinpoints toward the cell milieu as a significant determinant of the functional outcome of p73 isoforms. This functional controversy is particularly evident in the context of neoangiogenesis, in which TAp73 manifests a context-dependent dual role, suggesting that other modifiers in the cell milieu co-determine and thus impart its ultimate effect on the process (Sabapathy, 2015). Fourth, TAp73alpha shows a preference for genes with a distinct promoter architecture compared to the ones that specifically respond to TAp73beta, a finding that implies a C-terminus-based selectivity of TAp73 isoforms for target genes (Koeppel et al., 2011). These evidences overall suggest that p73-governed gene regulatory programs may be further orchestrated by indirect mechanisms that extend beyond their canonical role as transcriptional regulators, which compete for occupying gene promoters (Marabese et al., 2007).

In this section we provide compelling evidence to support that p73-regulated functions are a result of a sophisticated combination of at least three parameters: (a) the type of p73 isoforms, (b) the presence of p73 interacting partners in the cellular milieu, and (c) the intrinsic properties of promoters of the target genes. Tissues are characterized by distinct proteomes, hence the expression of the same p73 isoform in one tissue content could lead to functional diversification, since this isoform is able to form protein–protein interactions (PPIs) with a particular set of factors that is contained in one tissue but not in another. In addition, the C-terminus of p73 determines whether PPI occurs, because even if the interactor is present in the cell, the expressed p73 splice variant may not interact with it. Furthermore, p73 isoforms form protein complexes with several other key transcriptional regulators on target gene promoters to fine-tune their transcriptional regulation. Besides, some p73 isoforms appear to have non-transcriptional functions by interacting with proteins other than transcriptional regulators outside the nucleus (Tomasini et al., 2009; Vernole et al., 2009), suggesting that the subcellular localization of p73 isoforms can be an additional co-determinant of the p73 functional repertoire.

### The Characteristics of the p73 Protein Interactome

The p73 protein interactome is a decisive parameter of the tissue- and/or cell context-specific p73 activity. Several proteins have been described to physically associate with p73 isoforms, by recognizing the TA domain, the DBD domain, the OD or the C-terminus. These interactions usually take place in the nucleus and regulate the transactivation activity of p73 either positively or negatively. However, in some cases the interactions occur in the cytoplasm (Table 1). Another feature of the p73 interactome is that complex feedback loops can be generated among p73 and their binding partners. For example, PIR2 is a direct p73 target gene, and its protein product associates with DNp73 and promotes its proteosomal degradation (Sayan et al., 2010). Sp1 activates transcription from the P1 promoter of TP73 but also has the potential to form Sp1–TAp73 complexes, which can modulate Sp1 binding to corresponding elements on target gene promoters (Logotheti et al., 2010). Another candidate is the p73 transcriptional target NGFR (Logotheti et al., 2020b; Niklison-Chirou et al., 2020) that directly binds p73 isoforms at the DBD to facilitate their proteosomal degradation via chaperone-mediated autophagy (Nguyen et al., 2020).

**TABLE 1:** Interacting partners of p73 isoforms and their outcome on p73 function.

Protein interactor	P73 domain that mediates the PPI	Site of interaction	Outcome	References
MDM2	TA	Nuclear	Blocks p73 transcriptional activity by competing with p300/CBP binding without inducing proteolytic degradation	Bálint et al., 1999; Zeng et al., 1999
Pin1	C-terminus	Nuclear	Stabilizes p73, promotes conformational change of p73 and enhances its proapoptotic activity	Ozaki et al., 2010; Logotheti et al., 2013
YAP1	C-terminus (PPPPY)	Nuclear	p73-coactivator, potentiates p300-mediated acetylation of p73 and promotes stabilization by displacing Itch binding to p73	Ozaki et al., 2010; Logotheti et al., 2013
Itch	C-terminus (PPPPY)	Nuclear	Ubiquitination of p73 and subsequently promoting its proteasome-dependent degradation	Ozaki et al., 2010; Logotheti et al., 2013
FBXO45	C-terminus (SAM)	n.d.	Promotes the proteasome-dependent degradation of p73	Ozaki et al., 2010; Logotheti et al., 2013
PIASγ	C-terminus (OD)	Nuclear	Stabilizes p73 but together with SUMO-1 decreases functional activation of p73 by sumoylation	Ozaki et al., 2010; Logotheti et al., 2013
c-Abl	C-terminus (3K)	Nucleus	c-Abl-mediated phosphorylation of p73 induces p300-mediated acetylation, enhances interaction between Pin1 and p73 and therefore increases its stability and transcriptional as well as proapoptotic activity	Ozaki et al., 2010; Logotheti et al., 2013
NEDL2	C-terminus (PPPPY)	Cytoplasm	Promotes polyubiquitination of p73, stabilizes and enhances its transcriptional activity	Ozaki et al., 2010; Logotheti et al., 2013
JNK	n.d.	Nucleus	JNK-mediated phosphorylation of p73 promotes its stabilization, p300-mediated acetylation and transcriptional activity	Ozaki et al., 2010; Logotheti et al., 2013
IKK	DBD	Nucleus	Stabilizes p73 by inhibiting its polyubiquitination, enhances transcriptional activation and proapoptotic activity of p73	Ozaki et al., 2010
CDK complex	DBD, C-terminus (SAM)	Nucleus	Inhibits transcriptional activity of p73 by phosphorylation of Thr86	Gaiddon et al., 2003; Ozaki et al., 2010
PKA-Cβ (PRKACB)	N- and C-terminus	n.d.	Inhibits transcriptional and apoptotic activity of p73 by phosphorylation	Ozaki et al., 2010
HCK	N-terminus	Cytoplasm	Stabilizes cytoplasmic p73, inhibits transcriptional and apoptotic activity of p73	Ozaki et al., 2010
PLK1/PLK3	TA	Nucleus	Inhibits p73 transcriptional and apoptotic activity	Ozaki et al., 2010
p300	TA	Nucleus	Stabilizes p73 by acetylation at Lys321, Lys327, and Lys331, enhances p73 transcriptional and apoptotic activity	Ozaki et al., 2010; Logotheti et al., 2013
SIRT1	n.d.	n.d.	Inhibits transcriptional and apoptotic activity of p73	Ozaki et al., 2010
HIPK2	OD	Nucleus	Enhances the transcriptional activity of p73	Ozaki et al., 2010
amphiphysin IIb-1	C-terminus (3K)	Cytoplasm	Relocalizes p73 to the cytoplasm, inhibits transcriptional and apoptotic activity of p73	Kim et al., 2001; Ozaki et al., 2010
Wwox	C-terminus (PPPPY)	Cytoplasm	Tumor-suppressor; relocalizes p73 to the cytoplasm, inhibits the transcriptional activity of p73, p73 increases proapoptotic activity of Wwox	Ozaki et al., 2010; Logotheti et al., 2013
ASPP1/ASPP2	DBD	Nucleus	Selectively enhances proapoptotic function of p73	Ozaki et al., 2010
p19ras (HRAS)	DBD	Nucleus	Blocks MDM2-mediated transcriptional repression of p73 and led to the activation of p73	Ozaki et al., 2010
MM1	Extreme C-terminus	Nucleus	Selectively enhances transcriptional and growth-suppressing activity of p73	Ozaki et al., 2010; Logotheti et al., 2013
RanBPM	Extreme C-terminus	Nucleus	Stabilizes p73 by inhibiting its ubiquitination; enhances its transcriptional and proapoptotic activity	Ozaki et al., 2010; Logotheti et al., 2013
WT1	n.d.	Nucleus	Tumor-suppressor; inhibits transcriptional activity of p73	Ozaki et al., 2010
HCV core protein	Extreme C-terminus	Nucleus	Selectively inhibits the transacriptional and proapoptotic activity of p73	Ozaki et al., 2010
E4orf6	OD	n.d.	Inhibits transcriptional and proapoptotic activity of p73	Ozaki et al., 2010
CTF2 (CTF/NF-1)	DBD	Nucleus	Inhibits the sequence-specific DNA-binding activity of p73	Ozaki et al., 2010
BAG-1	n.d.	n.d.	Decreases expression of p73 and inhibits its transcriptional activity	Ozaki et al., 2010
TIP60	n.d.	Nucleus	Enhances MDM2 binding affinity to p73 and therefore inhibits its transcriptional and proapoptotic activity	Ozaki et al., 2010
PKP1	C-terminus (SAM)	Cytoplasm	n.d.	Neira et al., 2021
ETS2	C-terminus (SAM)	Nucleus	Forms a complex with DNp73, which directly activates ANGPT1 (angiogenesis and promoting tumor growth) and Tie2 (cell survival and proliferation) gene expression in tumor cells	Cam et al., 2020
NGFR	DBD	Cytoplasm	Inactivates p73 transcriptional activity by promoting its degradation	Nguyen et al., 2020
DGCR8	C-terminus (PPPPY)	Nucleus	Is predicted to interact with p73 and thereby influencing miRNA processing	Boominathan, 2010
GemC1	n.d.	Nucleus	Recruits p73 to E2F5 to selectively transactivate genes involved in multiciliogenesis as well as p73 itself	Lalioti et al., 2019
Sp1	n.d.	Nucleus	Prevents Sp1 binding to target promoter and subsequently its transcriptional activity	Koutsodontis et al., 2005; Racek et al., 2005
MCL1	OD	Nucleus	Inhibits p73 DNA binding and therefore inhibits its transcriptional activity	Widden et al., 2020
PIR2	n.d.	n.d.	Modulates p73 stability, alters TA/DNp73 ratio by promoting preferential degradation of DNp73	Sayan et al., 2010
Bub1	C-terminus (SAM)	Nucleus	TAp73 regulates SAC protein localization and activities, deregulation of p73 can alter mitotic checkpoint abilities and induce polyploidy	Tomasini et al., 2009; Vernole et al., 2009
Bub3	C-terminus (SAM)	Nucleus	Deregulation of p73 can alter mitotic checkpoint abilities and induce polyploidy	Vernole et al., 2009
BubR1	C-terminus	Nucleus	TAp73 but not DNp73 potentiates BubR1 activity, regulates SAC protein localization and activities	Tomasini et al., 2009
HIF-1α	n.d.	n.d.	p73 affects HIF-1α protein stability and subsequently ubiquitin-dependent proteasomal degradation in an oxygen-independent manner	Amelio et al., 2015
FLASH	C-terminus	n.d.	Regulation of histone gene transcription	De Cola et al., 2012
Cul4A-DDB1	n.d.	Nucleus	Inhibits transcriptional activity of p73	Malatesta et al., 2013
SUMO-1	Extreme C-terminus	Nucleus	Enhances proteosomal degradation of TAp73α	Logotheti et al., 2013
RACK1	Extreme C-terminus	Nucleus	Downregulation of apoptotic targets and inhibition of TAp73α-mediated apoptosis	Logotheti et al., 2013
PTEN	C-terminus (SAM)	Nucleus	Enhances the transcriptional activation of apoptotic genes	Logotheti et al., 2013
UFD2A	C-terminus (SAM)	Nucleus	Ubiquitination of p73 and subsequently promoting its proteasome-dependent degradation	Logotheti et al., 2013

*n.d., not determined.*

**FIGURE 2:**
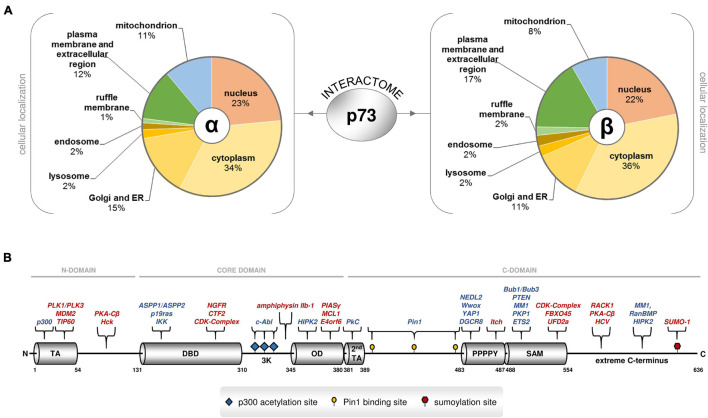
Several proteins physically associate with p73 isoforms inside and outside the nucleus. **(A)** Analysis of the cellular localization of the p73alpha and beta interactome based on high-throughput coIP-MS screening in Saos-2 cells overexpressing TAp73alpha, ΔTAp73alpha, TAp73beta, or ΔTAp73beta. Protein annotation and subcellular distribution was retrieved from UniProt and DAVID GO. **(B)** The TP73 gene encodes a variety of functional motifs and crucial amino acid residues in the N-terminus, the core domain, and the C-terminus that serve as selective interacting surfaces for the p73 proteome. The C-terminus is particularly enriched in such protein-binding sites. Each p73 isoform contains unique combinations of these protein-binding sites. Annotations: TA transactivation domain, DBD DNA binding domain, OD oligomerization domain, 3K p300 acetylation sites, 2nd TA second transactivation domain, SAM sterile alpha motif, extreme: extreme carboxy terminus. The literature-curated protein interactors that recognize the corresponding motifs and residues are also depicted. The proteins with inhibitory effect on p73 stability or activity are highlighted in red.

Several p73 protein binding partners induce post-translational modification, proteolytic degradation, phosphorylation-dependent activation or inhibition, acetylation or gene target co-regulation, often in a p73 C-terminus-dependent manner (Logotheti et al., 2013). Other proteins bind to p73 isoforms and retain them in the cytoplasm, thereby interfering with p73-mediated transcription of its target genes. Moreover, p73 isoforms can interact with non-transcriptional regulators in the nucleus, intimating that they are also involved in processes beyond gene transactivation. For example, TAp73 regulate the spindle assembly checkpoint (SAC) during mitosis and meiosis via physical interaction with components of the SAC complex, such as Bub1, Bub3 and BubR1, the inhibitor of anaphase-promoting complex protein Cdc20, regulating their proper localization (Tomasini et al., 2009). TAp73alpha interacts with the kinetochore-related proteins Bub1 and Bub3, which leads to the alteration of mitotic checkpoint abilities and induction of polyploidy. This association is specific for TAp73alpha but not p53 or any of the other p73 forms (Vernole et al., 2009). Using computational approaches, a previous study predicted that some p73s can interact with DGCR8 (Boominathan, 2010), a nucleus-localized, highly conserved component of the miRNA processing machinery which binds pri-miRNA to stabilize it for processing by Yeom et al. (2006). Importantly, DGCR8 is a miRNA-processing protein that is indispensable for miRNA maturation, and its ablation leads to early developmental arrest due to the lack of maturation of pre-miRNA products (Wang et al., 2007). It is therefore possible that p73alpha and p73beta isoforms may crosstalk with the miRNA processing machinery to control the quality and quantity of mature miRNA populations by physically associating with DGCR8. Considering that p73 isoforms can exhibit cytoplasmic localization (Dobbelstein et al., 2005; Nekulová et al., 2010), this raises the possibility of additional functions beyond transcription, in subcellular organelles outside the nucleus. In agreement with this notion, the results of our coIP-MS analyses in Saos-2 cells in which distinct p73 isoforms were exogenously added demonstrated that p73 variants have the ability to bind to proteins in the ER, Golgi apparatus, mitochondria, and endosome. Of particular interest, p73alpha and p73beta can bind to proteins associated with the ruffle membrane, plasma membrane and extracellular region (Figure 2A).

### The C-Terminus as the Basis for Protein–Protein Interaction Selectivity

While the N-terminus of TP73 has provided a first rule of thumb for the functional characterization and classification of the many gene products based on the presence of TA and the corresponding ability to activate transcription of gene targets, the C-terminus inevitably represents the most variable region of the TP73 gene. A total of eight alternative splicing events in the 3′ end and one alternative termination event in a portion of exon 13 generate 10 different versions of the C-terminus. These carboxy-terminal tails are combined with a main core domain that exerts DNA binding and oligomerization ability, and an N-terminus with or without canonical transactivation activity, giving rise to more than twenty gene products (Logotheti et al., 2013). The significance of the C-terminus for p73-mediated functions is underscored by the recently described Trp73^d13/d13^ mice, in which a switch from the α to β form through knockout of exon 13 results in distinct phenotypic abnormalities that do not overlap with those of other p73 knockout mice (Niklison-Chirou et al., 2020). The C-terminus is a highly active region, both in terms of binding to proteins and DNA. First of all, it carries an electrostatic charge that differs significantly between p73 variants and affects promoter binding and gene transactivation. More importantly, it is particularly enriched in unique motifs and crucial amino acid residues that serve as protein-interacting surfaces. Because of the numerous alternative splicing events in the 3′ end, each C-terminal variant bears its own combination of these motifs. The versatility of the C-terminus can be particularly associated with the many different biological activities of p73 isoforms, as each isoform carries its own unique combinations of functional domains and motifs that are recognized by distinct groups of protein interactors. Overall, the C-terminus may act as a platform for the selection of p73 PPIs that can play a decisive role in both the nature of target genes and the degree of activation, possibly via differential interactions with regulatory proteins.

The functional domains and residues of the C-terminus have been highlighted previously and are shown, along with their corresponding protein interactors in Figure 2B. First, the carboxy-terminal region 380–513 is spanned by Glu/Pro-rich and Pro-rich regions that exhibit transactivation activity. In particular, it entails a glutamine/proline-rich domain within amino residues 381–399 that is phosphorylated by PKCα2 and PKCβ in Ser388, and regulates genes involved in cell cycle progression; three crucial pS/pT-P motifs at residues 412, 442 and 482, which are specifically recognized by the propyl isomerase Pin1, a chaperone that catalyzes the isomerization of peptidyl-propyl bond from *cis*- to *trans*-conformation and regulates transactivation efficiency, stability and subcellular localization; and a highly conserved PPPPY motif in residues 483–491 that is specifically targeted by proteins bearing a WW domain, causing them to develop PPPPY-WW-mediated PPIs. The WW-containing interactors of p73 are (a) the yes-associated protein YAP, a phosphoprotein that interacts with a non-receptor Src tyrosine kinase encoded by the c-yes protooncogene, (b) NEDD4-like ubiquitin protein ligase 2 (NEDL2), (c) cytoplasmic tumor suppressor oxidoreductase (Wwox), and (d) E3 ubiquitin ligase Itch. YAP, NEDL2, and Wwox enhance the transcriptional activity of p73, while Itch is a YAP antagonist, that leads to p73 ubiquitination and degradation and impairs transcriptional activity (Logotheti et al., 2013). DGCR8 was also predicted *in silico* to possess a WW-domain, which may interact with the PPPPY motif of the C-terminal domains of p73α and p73β (Boominathan, 2010).

Additional interacting surfaces downstream of PPPPY render TAp73α susceptible to mediators of ubiquitin-proteasome degradation, as well as other effectors of p73 stability and activity. In detail, the SAM extends between residues 487–554 and is recognized by PTEN, an inducer, and by UFD2A and FBXO45, two attenuators of TAp73α transactivation efficacy. The SAM domain can also bind to the N terminus of MDM2 (Neira et al., 2019). Plakophilin 1 (PKP1), a component of desmosomes, which are key structural components for cell-cell adhesion, also recognizes the SAM domain (Neira et al., 2021). Moreover, residues 555–636, representing the extreme C-terminus of p73 alpha and comprising four conserved sequence motifs (Logotheti et al., 2013), are recognized by RanBPM, a cellular interactor of the nuclear-cytoplasmic transport protein Ran, which stabilizes TAp73alpha and enhances its transactivation activity, most likely by masking C-terminal lysine residues that could be the sites for ubiquitin ligation and/or disrupting the interaction of TAp73alpha with unknown proteins required for ubiquitin-mediated proteolysis. Another protein that physically interacts with TAp73alpha via the extreme C-terminus is MM1, a c-myc binding protein. Upon binding to p73, it selectively enhances transcription of specific p73 target genes, thereby potentiating growth suppression. MM1 antagonizes the inhibitory effect of c-myc on the extreme C-terminal domain-containing TAp73alpha isoform by preventing the c-myc-p73alpha interaction and/or directly binding to c-myc to inhibit its activity. In addition, the receptor for activated C kinase RACK1 physically interacts through the extreme C-terminus, leading to the downregulation of apoptotic targets and inhibition of TAp73alpha-mediated apoptosis. Finally, small ubiquitin-like modifier 1 (SUMO-1), binds to TAp73alpha through a covalent modification of Lys627, making this isoform more susceptible to proteosomal degradation [reviewed in Logotheti et al. (2013)].

These motifs and residues appear to be important in “finalizing” the effects of the major p73 interactors by recruiting their essential co-factors. For example, it is well-established that TAp73alpha can bind to MDM2 via its OD, but unlike its p53 sibling, this interaction does not lead to protein degradation of p73 (Bálint et al., 1999; Zeng et al., 1999). Only in the presence of Itch, which recognizes PPPPY motifs, can p73 protein degradation finally occur (Bálint et al., 1999; Zeng et al., 1999; Rossi et al., 2005). In a similar manner, c-abl induces phosphorylation of p73 at the NH2-terminus, but additional p73 interactors selective for the C-terminus are needed to enable p73-mediated apoptosis. DNA damage activates c-Abl, which phosphorylates TAp73 directly at site Tyr99 and indirectly, via p38, at Pin1-binding sites 412, 442, and 482. Subsequently, Pin1 targets the phosphorylated residues and catalyzes the conformational change at TAp73. The c-abl phosphorylated YAP1 binds to the PPPPY motif and attracts p300, which in turn acetylates a 3K motif downstream of the OD domain. In this state, the complex selectively binds to transactivate apoptotic versus cell cycle arrest targets (Logotheti et al., 2013). Several of the abovementioned motifs and residues, together with their corresponding interactors, have been conserved within vertebrate clades where p73 has split from its p53/p63/p73-hybrid ancestor. The patterns of co-conservation of the C-terminal motifs and corresponding protein interactors suggest that the corresponding PPIs are required for p73 functions (Logotheti et al., 2013). It is therefore possible that the increase in the number of splice variants reflects the enhanced potential of p73 to evolve PPIs that can support its expanding functional repertoire.

### The Promoter Architecture of Direct and Indirect p73 Target Genes

**FIGURE 3:**
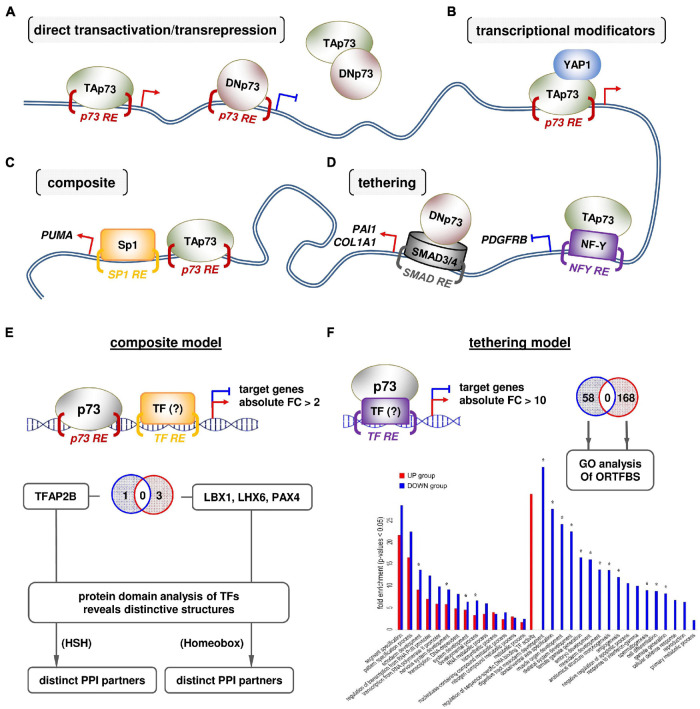
An interplay of gene promoter architecture with p73 binding partners orchestrates the functional diversity of the p73 isoforms. **(A)** Typical transcription factor-based and **(B–D)** PPI-based modes of genomic action of p73 isoforms. **(E,F)** Computational prediction of p73 PPIs relative to the promoter architecture by meta-analysis of extrapolated p73 ChIP-seq and Mel.DNp73 microarray data (Koeppel et al., 2011; Steder et al., 2013). **(E)** *Composite scenario:* the overrepresentation (*Z*-score) of transcription factor binding sites (ORTFBS) in the DNp73-responsive gene groups (using Pscan software): (A1) >2× over-expressed (OE); (A2) >10× OE; (B1) >2× underexpressed (UE); (B2) >10× UE. The groups A1 and B1 were integrated with ChIP-seq data on p73 binding sites. The distance of the p73REs from the selected TFBS in A1 and B1 groups was calculated by Pscan using the “Report Occurrences” function with a PWM-threshold of 0.75. The distance score was set as 1 if the distance was within a range of 15–250 bp and to 0 otherwise. Afterward, the relative number of close TFBS within each group was calculated by setting up the ratio of positive distance hits to the total number of genes in the respective group. This ratio in each group (A1, B1) was compared to the ratio of the same TFBS versus all genes in the microarray to calculate the enrichment of close TFBS. The enriched TFBS were significantly close to the respective p73RE in group B1 (estimated by *t*-test), whereas a significant enrichment in group A1 could only be found for one TF. **(F)** *Tethering scenario:* factors that bind DNp73 were predicted using the A2 and B2 groups of DNp73-responsive genes. The number of TFs recognizing the enriched ORTFBS for A2 and B2 were 58 and 158, respectively. The functions in which TFs are involved were predicted by GO-term analysis.

The p73 isoforms show complex patterns of interaction with target gene promoters in addition to their canonical mode of gene transactivation/transrepression (Figure 3A). C-terminal variant-specific transcriptional responses have been described previously for TAp73alpha and TAp73beta. The genes responding to TAp73alpha have different binding patterns and gene promoter architectures than TAp73beta-responsive ones. Gene promoters occupied by TAp73alpha are enriched in the AP1 motif, which can bind to Jun/Fos family heterodimers, and this is associated with more frequent upregulation of genes with AP1 motif in comparison to genes lacking this motif. In contrast, promoters occupied by TAp73beta do not exhibit this motif and do not show similar activations of AP1-responsive genes (Koeppel et al., 2011). TAp73 can physically interact with c-Jun of the AP-1 complex on target gene promoters via its carboxyl-terminal region (Subramanian et al., 2015). Taken together, these data underscore a p73 isoform-specific selectivity of target genes that is shaped not only by their intrinsic promoter characteristics but also by PPIs between p73forms and other “extrinsic” transcription factors on these promoters. Based on these findings, we postulate that the interplay of gene promoter architecture with p73 binding partners orchestrates the functional diversity of p73 family members. Target genes may bear typical p73-responsive elements (RE), but there are also cases of indirect p73 targets which lack them. Several p73 isoforms physically associate with other transcriptional regulators at target gene promoters and modify transactivation both in a direct p73-RE-dependent and an indirect p73RE-independent manner (Racek et al., 2005; Beitzinger et al., 2006; Buhlmann et al., 2008). First scenario, p73 isoforms interact with co-regulators at the promoters of p73-responsive genes (Figure 3B), which induce post-translational modifications to p73 proteins and fine-tune target genes that are eventually transactivated. Such co-activator examples are YAP1 and Pin, which form complexes with TAp73 in response to DNA damage to favor transcription of p73-responsive pro-apoptotic genes (Logotheti et al., 2013). Second, p73 isoforms tether on transcription factors bound to promoters lacking a typical p73RE and modulate their transcriptional activity. For instance, TAp73 interacts with NF-Y bound on the PDGFRB promoter and turns off gene expression, whereas DNp73 interacts with SMAD3/4 bound to SMAD-responsive elements and potentiates activation of the PAI1 and COL1A1 genes (Engelmann et al., 2015) (Figure 3C). Similarly, ΔNp73 transcriptionally upregulates both ANGPT1 and Tie2 through conserved ETS-binding sites by interacting with ETS2, resulting in forced angiogenesis and survival of glioblastoma (Cam et al., 2020). Third, p73 isoforms act in a composite manner both by direct binding to p73REs and by physical interaction with transcription factors that bind to adjacent sites. An example is the cooperative activation of PUMA by TAp73beta and Sp1, both of which associate with neighboring responsive elements at the PUMA promoter and physically interact with each other (Ming et al., 2008) (Figure 3D).

### Using the Promoter Architecture of p73-Responsive Genes as ‘Footprint’ for Predicting Novel, Functionally Relevant p73 Protein–Protein Interactions

To further explore the idea that the architecture of gene promoters related to p73 PPIs is important for the function of p73 isoforms, we used microarray transcriptomics data that we have produced previously (Steder et al., 2013). Ectopic stable expression of DNp73 in the low-invasive SK-Mel-29 cells causes whole-transcriptome changes and supports metastasis-initiating phenotypes, such as stemness, EMT and invasion, and de-differentiation (Steder et al., 2013; Meier et al., 2016; Fürst et al., 2019). If the hypothesis that the effect of DNp73 on these cancer cell phenotypes is achieved through PPI of DNp73 with nodal molecules on gene promoters is solid, then the architecture of the promoters of the genes that are deregulated upon DNp73 overexpression can serve as a ‘footprint’ of these PP-interactions. In detail, the DNp73-responsive transcriptome will be enriched of genes which have binding sites for specific and functionally-coherent interactors in their promoter regions. Hence, we sought to meta-analyze this transcriptome data to predict novel, functionally coherent interactors of DNp73 in the promoter regions of these genes. To this end, we integrated the high-throughput mRNAs array of stable SK-Mel-29.DNp73 clones versus its mock counterpart (Steder et al., 2013) with p73 ChIP-seq data (Koeppel et al., 2011) and searched for overrepresentations of binding motifs for transcriptional regulators either near p73REs (composite scenario) or independent of p73REs (tethering scenario) in the promoters of DNp73-responsive genes. For example, when DNp73 interacts with the transcription factor TF1, the upregulated and downregulated genes in the transcriptome will have over-represented binding sites for TF1. In the case of a composite mechanism, p73REs will be found in close proximity (up to 250 bps) to TF1 REs (Koeppel et al., 2011) to allow physical association between DNp73-TF1. On the other hand, in case of a tethering mechanism, where DNp73 binds independently of p73Res to, e.g., TF2, many significantly up- or downregulated DNp73-responsive genes (more than 10-fold increase) without p73REs would instead have overrepresented binding sites for TF2. As consequence, TF1 and TF2 should have functional relevance to the resulting cell phenotype. With this approach, we were able to predict potential binding partners of DNp73. Our results in detail are:

(a) *Composite scenario:* we found a clear enrichment of TFBS for LBX1, LHX6, and PAX4 in close proximity to p73REs in genes that are downregulated in response to DNp73, whereas in the case of DNp73-upregulated genes, TFAP2B binding sites were significantly enriched. Overall, we predicted that DNp73 binds adjacent to responsive elements for LHX6, PAX4, and/or LBX1 as well as TFAP2B transcription factors and down- or upregulates the corresponding genes, suggesting that these proteins are candidates for p73 co-regulators. Three striking observations were made by this analysis: (Logotheti et al., 2013) candidate p73 co-regulators for downregulated genes differs from predicted p73 co-regulators for upregulated genes. The complete lack of overlap implies a ‘deterministic trend’ of DNp73 to develop highly “selective” PPIs with the candidate co-regulators; (Belyi and Levine, 2009) candidate p73 co-regulators are important neurodifferentiation/neurodevelopment factors, which suggests the involvement of neurodifferentiation programs in the effect of DNp73 on cancer aggressiveness; and (Belyi et al., 2010) the likely co-regulators for downregulated genes have different structures than those for upregulated genes: LHX6, PAX4, and LBX1 are homeobox proteins, whereas TFAP2B is a basic helix-loop-helix protein. These instances intriguingly imply that DNp73 can select interaction candidates at gene promoters based on structure (Figure 3E).

(*b*) *Tethering scenario:* we checked ORTFBS in DNp73 genes that are highly increased (A2, >10-fold) or decreased (B2, <10-fold) and found non-overlapping groups of ORTFBS between both groups (A2 vs. B2) (Figure 3F). In A2, genes with binding sites for 58 TFs were significantly enriched, while in B2, 158 TFs behaved in this way. GO-term analysis of the ORTFs of each group revealed that these ORTFs tend to be involved in cellular and embryonic developmental/differentiation processes (Figure 3F, annotated with ^∗^) in a non-overlapping manner.

In summary, the interplay between p73 isoforms with different C-termini, their interacting partners, and the architecture of the target gene promoter supports a high degree of heterogeneity in the mechanisms underlying tissue-specific p73-driven functions in a variety of organ systems including immunity, neurodevelopment and reproduction. Such sophisticated mechanistical patterns may explain, at least partially, the different and sometimes opposing results across different cell contents and experimental settings. Dysregulation of one or more of the above parameters in tumors could lead to tumor initiation and progression by, at least in some cases, reactivating p73-regulated differentiation programs in a spatiotemporally inappropriate manner.

## Conclusion and Future Perspectives

The main body of research on TP73 has been initially performed in the cancer setting, due to its functional and structural similarity with the tumor-suppressor TP53. In cancer, the networks controlled by the typically anti-oncogenic TAp73 isoforms offer functional redundancy to the intricate circuitries regulated by p53, through activating fully or partially overlapping pathways, which can circumvent blocks attributed to mutations in TP53 or its downstream effectors (Logotheti et al., 2019). Nevertheless, it is now clear that TP73 with the complexity of isoforms is a key regulator of an own unique and wide range of biological aspects in embryonic development, differentiation, homeostasis, and immune response. This pleiotropy has rejuvenated the interest in p73 as a therapeutic target for the management of not only cancer, but also several other complex diseases, such as neurodevelopmental disorders, COPD (Nemajerova and Moll, 2019), sterility (Inoue et al., 2014), metabolic disorders (Jia et al., 2018), and autoimmune disease susceptibility (Ren et al., 2020). Several common unifying themes can be recognized among the diversity of physiological and oncogenic p73 functions, such as the ability of p73 isoforms to act as transcriptional master regulators of motile multiciliogenesis underlying the disparate p73KO phenotypes of airway infections and female and male infertility (Nemajerova and Moll, 2019). Moreover, to determine tissue architecture, through regulating cell adhesion, cytoskeleton dynamics and planar cell polarity, which is essential for the organization and homeostasis of various complex microenvironments, like the neurogenic niche, reproductive organs, respiratory epithelium, or vascular network (Maeso-Alonso et al., 2021).

In view of recent findings, the potential of p73 isoforms to modulate cancer initiation and progression might not be independent from the physiological functions of TP73. Instead, it could reflect the recapitulation of the same p73-regulated developmental/tissue homeostasis pathways within the cancer cell content. Specifically, a new idea proposes that metastatic transcriptional programs arise from *de novo* combinatorial activation of multiple distinct and developmentally distant transcriptional modules (Rodrigues et al., 2018). Although mutations in tumor suppressors and oncogenes predominate during tumor initiation, cancer cells become metastatic at progression stages, often by hijacking gene expression programs of normal embryonic development and reactivating them outside their physiological context. In support of this notion, we have recently shown that p73-driven neurodevelopmental pathways are co-opted in cancer cells to promote the acquisition of neurogenic features in melanoma cells via production of secreted neurotrophins, such as NGF and BDNF (Logotheti et al., 2020b). This process plausibly facilitates cancer cells to gain neurogenic potential and communicate with neuronal cells, providing overall selective advantages for tumors. More intriguingly, specific factors of the cancer cell secretome force a variety of surrounding cells in the TME, including fibroblasts, endothelial cells, bone marrow-derived cells, immune cells, and neurons to integrate into the stroma, where their activities are redirected to benefit cancer cell progression. Given that BDNF and NGF are recognizable by both, neuronal cells and immune cells, an appealing hypothesis is that reactivation of p73 neurodevelopmental networks in cancer cells leading to neurotrophin production and secretion may establish a complex cancer–neuroimmune crosstalk in the TME that alters the dynamics of cellular interactions toward an unfavorable prognosis (Logotheti et al., 2020b).

**FIGURE 4:**
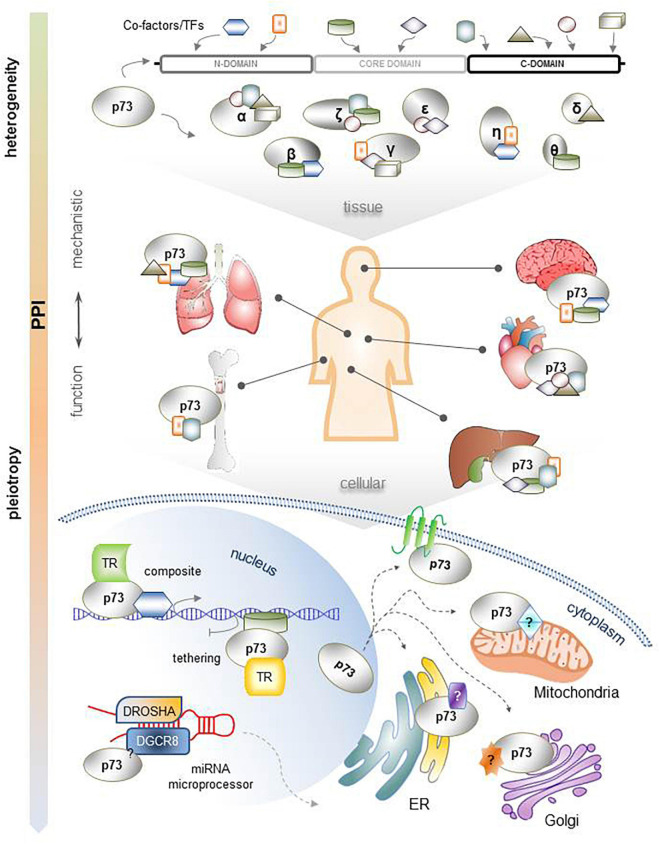
Comprehensive illustration of the mechanistic heterogeneity that supports the functional pleiotropy of TP73. Each p73 isoform has a unique combination of interacting motifs and residues that can develop distinct PPIs. This is particularly important for their function across several organ systems, which present tissue-specific protein contents. The resulting complexes of p73 isoforms with their protein binding partners interact with gene promoters according to composite or tethering mechanisms of gene transcription regulation; are integrated in other multi-protein complexes within the nucleus, such as the miRNA processing complex, to control miRNA maturation; or are localized in the cytoplasm, possibly affecting the function of several subcellular organelles and/or the plasma membrane.

The functional pleiotropy of TP73 assumes existence of mechanistic heterogeneity that extends beyond its typical transactivation mode of action. The PPI-mode of action of TP73 can provide a basis for its multifunctionality and tissue specificity. We propose that multiple p73 isoforms can establish PPIs with many proteins via several motifs in the N-terminus, core domain, and C-terminus, with the C-terminus being particularly enriched in protein binding motifs and residues. In different tissues, p73 isoforms with different C-termini may have different binding partners that can be recognized by the appropriate interacting regions of 73, causing the formation of a variety of p73 coregulator complexes. The localization of these complexes can also lead to functional diversification. Thus, p73-containing complexes within the nucleus select direct and indirect p73 target genes and regulate them via composite and tethering mechanisms based on a specified promoter architecture. In addition, p73 isoforms may be able to bind to other proteins as transcriptional regulators, such as the miRNA processing complex and SAC. p73 isoforms also physically associate outside the nucleus with proteins localized in a number of subcellular organelles. The ER- and Golgi-related interactome reflects to some extent the well-established tendency of TP73 gene products to undergo post-translational modifications. However, their potential to interact directly with proteins in intracellular compartments, such as the cell membrane, lysosomes, endosomes, and mitochondria might indicate novel, non-transcription-mediated functions of p73 isoforms that are worth unveiling in the future. A comprehensive scheme of the heterogeneity of mechanisms supporting p73 functional pleiotropy and diversity is shown in Figure 4. The proposed mechanistic models could explain, at least in part, the different and sometimes conflicting results in different cell contents and experimental settings. Dysregulation of one or more of the above parameters in tumors could lead to cancer progression by reactivating p73-regulated differentiation programs in a spatiotemporally inappropriate manner. Moreover, miRNAs that target and inhibit p73 mRNA at the post-transcriptional level [extensively described in Logotheti et al. (2019)], as well as several protein modificators of the activity and stability of p73 protein in the post-translational label [reviewed in detail in Conforti et al. (2012)] create additional layers of complexity in the mechanisms underlying the p73-mediated functions.

From the therapeutic perspective, these new insights provide a roadmap for efficient and selective manipulation of p73 isoforms toward precision medicine. On the shoulders of structural systems pharmacology (Duran-Frigola et al., 2013; Xie et al., 2014), these mechanisms can be translated into personalized solutions against various complex diseases associated with p73 dysregulation. By applying relative structure-based computational pipelines, which we have recently successfully implemented to design strategies against the metastatic interactome of other key transcription factors such as E2F1 (Babushok et al., 2015; Logotheti et al., 2020a), the p73 isoform-coregulator complexes causally associated with pathological conditions can be identified and 3D models generated to reveal their interacting interfaces. Subsequently, structure-based pharmacophore modeling can be used to identify potential inhibitors that disrupt these PPIs and dissociate the pathological p73 coregulator complexes of interest by destabilizing the bonds at the sites of their physical association. These predicted inhibitors with prognosticating effect can become part of drug discovery programs for the development of next generation p73-based targeted therapeutics.

## Author Contributions

SL and BP conceived the review and took the lead in writing. SM developed bioinformatics pipelines and analyzed high-throughput data. CR conducted CoIP-MS and data analysis. NM, CR, and SL performed literature search. AS, SM, and SL crafted the illustrations. All authors contributed to and approved the final manuscript.

## Conflict of Interest

The authors declare that the research was conducted in the absence of any commercial or financial relationships that could be construed as a potential conflict of interest.

## Publisher’s Note

All claims expressed in this article are solely those of the authors and do not necessarily represent those of their affiliated organizations, or those of the publisher, the editors and the reviewers. Any product that may be evaluated in this article, or claim that may be made by its manufacturer, is not guaranteed or endorsed by the publisher.
